# Novel Polymeric Hybrid Nanocarrier for Curcumin and Survivin shRNA Co-delivery Augments Tumor Penetration and Promotes Synergistic Tumor Suppression

**DOI:** 10.3389/fchem.2020.00762

**Published:** 2020-09-29

**Authors:** Bei Xu, Wen Zhou, Lizhi Cheng, Yang Zhou, Aiping Fang, Chaohui Jin, Jun Zeng, Xiangrong Song, Xia Guo

**Affiliations:** ^1^Department of Pediatric Hematology/Oncology, Key Laboratory of Birth Defect and Related Disorders of Women and Children (Sichuan University), Ministry of Education, West China Second University Hospital, Sichuan University, Chengdu, China; ^2^Department of Otolaryngology, Tongji Medical College, Union Hospital, Huazhong University of Science and Technology, Wuhan, China; ^3^Department of Clinical Laboratory, Mianyang Central Hospital, Mianyang, China; ^4^State Key Laboratory of Biotherapy and Cancer Center, West China Hospital, Sichuan University, Chengdu, China

**Keywords:** curcumin, survivin shRNA, co-delivery, tumor penetration, nuclear targeting

## Abstract

A major barrier for co-delivery of gene medicine with small molecular chemotherapeutic drugs in solid tumors is the inadequate tumor penetration and transfection. In this study, a novel polymeric nanocarrier with integrated properties of tumor penetration, nuclear targeting, and pH-responsive features was designed, and further used to achieve the synergistic anti-tumor effect of curcumin (CUR) and survivin shRNA (pSUR). The polymeric hybrid nanocarrier was constructed from the FDA-approved polymer PLGA and a novel conjugated triblock polymer W5R4K-PEG_2K_-PHIS (WPH). CUR and pSUR were simultaneously encapsulated in the dual-drug-loaded nanoparticles (CUR/pSUR-NPs) by a modified double-emulsion solvent evaporation (W/O/W) method. The obtained nanoparticles exhibited better pharmaceutical properties with a uniform spherical morphology and sustained release manners of CUR and pSUR. Excellent features including preferable cellular uptake, efficient endosomal escape, enhanced tumor penetration, and elevated transfection efficiency were further proven. Additionally, a markedly enhanced anti-tumor efficacy for CUR/shRNA-NPs was achieved on SKOV-3 and Hela cells. The synergistic anti-tumor effect involved the inhibition of tumor cell proliferation, induction of cell apoptosis, and the activation of caspase-3 pathways. This work sets up an innovative co-delivery nanosystem to suppress tumor growth, contributing to the development of a comprehensive nanoparticulate strategy for future clinical applications.

## Introduction

A combination of multiple different therapeutic strategies with synergistic effects have been common practice for cancer therapy. Particularly, the co-administration of chemotherapy and gene therapy has shown great promise in achieving higher efficacy and potency with reduced toxicity (Chen et al., 2019; Meng et al., 2020). Curcumin, one of most commonly used chemotherapeutic drugs, suppresses the initiation, progression, and metastasis of many kinds of cancers. In addition, it appears to inhibit carcinogenesis, affecting angiogenesis, and tumor growth (Hamzehzadeh et al., 2018). Survivin is a member of the family of inhibitors of apoptosis proteins. It is selectively overexpressed in tumors, but not in normal tissues (Peery et al., 2017). Survivin expression is essential for cancer cell survival and survivin targeting has been envisaged as a novel therapeutic strategy (Shamsabadi et al., 2016; Khan et al., 2017; Frassanito et al., 2019). Inhibiting survivin's function or downregulating its expression results in spontaneous apoptosis (Peery et al., 2017). Herein, a new combination consisting of survivin short hairpin RNA (pSUR) and CUR was designed. We hypothesized that the combination of CUR and pSUR would achieve a synergistic or combining effect in the treatment of solid cancer.

Nanomedicine has been extensively engineered to enhance the anti-tumor efficacy of therapeutic agents (Afsharzadeh et al., 2018; Islam et al., 2018; Kong et al., 2019; Tao et al., 2019; Tang et al., 2020), especially for the gene/chemo-therapy combination. Unfortunately, due to inadequate penetration and gene transfection in solid tumors, it only achieves modest therapeutic efficacy. Many strategies have been utilized to improve the nanomedicines' tumor penetration, mainly by optimizing nanoparticle properties (sizes, shapes, and charges) and modulating tumor microenvironments (Shi et al., 2017; Sun et al., 2017). However, due to the unchanged passive diffusion of bulky nanomedicines against the interstitial fluid pressure gradient, the efficiency usually remains unsatisfactory (Ding et al., 2019). Transcellular transport that actively transports nanoparticles through cells by cell-penetrating peptides might be a universal approach for tumor penetration enhancement (Ding et al., 2019). For instance, iRGD exhibiting increased penetration ability and improved tumors' accumulation, has been broadly applied in various nanoparticles (Wu et al., 2019). However, its application might be limited by the expression of integrin and NRP-1/2 on endothelial cells and/or specific tumor cells, and it has no potential for nuclear targeting delivery, probably leading to inefficient gene expression.

Cyclic peptides containing arginine (W) and tryptophan (R) are found to be appropriate for cell-penetrating and have nuclear targeting properties (Mandal et al., 2011). For instance, synthetic cyclic peptides [WR]4 and [WR]5 had been applied to enhance the cellular uptake of phosphopeptides, doxorubicin, and anti-HIV drugs, with the potential of intracellular nuclear targeting delivery (Oh et al., 2014). The rigidity in these peptides can enhance the cell penetrating property (Nguyen et al., 2010; Lättig-Tünnemann et al., 2011). Additionally, because the cyclization improves resistance to proteolytic degradation, these peptides are expected to be more stable toward human serum than linear peptides (Nguyen et al., 2010). Despite these advantages, the application of these cyclic peptides in drug/gene delivery polymeric nanosystems still remains unexplored. Considering this, the cyclic peptides [W5R4]K (tryptophan, K) were selected in this work, and their potential of tumor penetration and nuclear targeting conjugated to nanoparticulate formulation were first evaluated.

To enhance the anti-cancer efficacy of current nanosystems, another greatly indispensable factor is to achieve a fast drug/gene release at tumor sites. The design of pH-sensitive nanoformulations has been regarded as a potent strategy for drug/gene selective release, owing to the typical pH in the interstitium of solid tumors (pH 5.7–7.8) and in the endo/lysosome of tumor cells (pH 5.0–6.0) (Li et al., 2015; Gao et al., 2017; Zhu et al., 2018). Otherwise, the utilization of pH-dependent mechanisms can also facilitate the nanosystems' escape from endo/lysosomal trafficking pathways into cytosol (Wilson et al., 2013). Among various pH-sensitive biomaterials, Poly (L-histidine) (PHIS) has been harnessed as an excellent candidate. Its imidazole ring (pKa 6.0–7.0) has lone pairs of electrons on the unsaturated nitrogen, which endow it with pH-dependent amphoteric properties (Lee et al., 2007; Radovic-Moreno et al., 2012; Qiu et al., 2014). Moreover, the protonated PHIS can interact with anionic phospholipids composing the endosomal compartments and result in appreciable endolysosomal membrane disruption (Lee et al., 2005). Thus, PHIS was selected in our work to establish the perfect pH-sensitive polymeric carriers.

**Figure 1:**
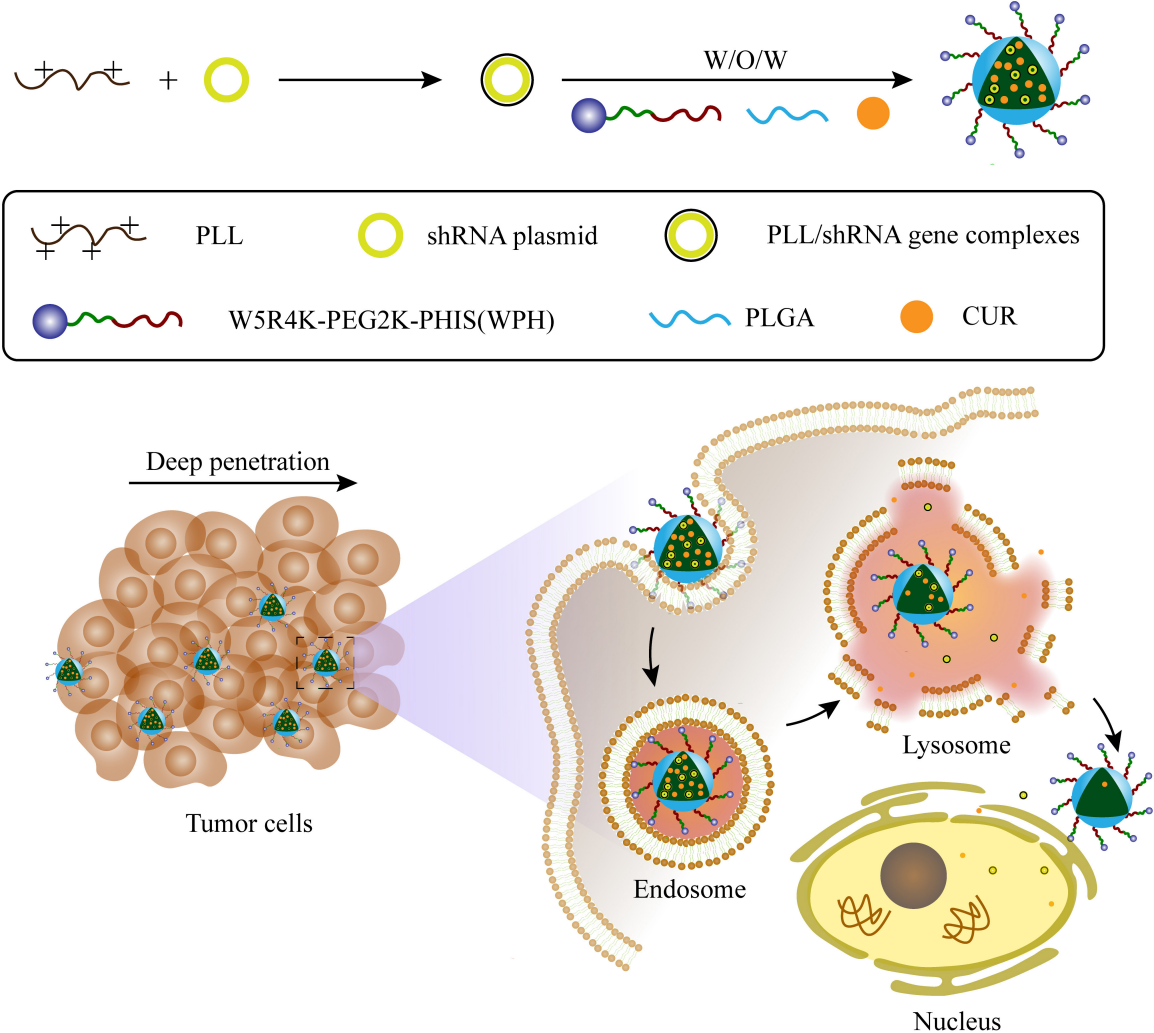
Schematic illustration of the preparation for CUR/pSUR-NPs and the proposed mechanism of CUR/pSUR-NPs including superior tumor penetration, pH-responsive features, as well as the properties of nuclear targeting.

Herein, we aimed to synthesize an amphiphilic copolymer that consisted of [W5R4]K, poly(ethylene glycol), and poly(L-histidine) (WRK-PEG-PHIS, WPH), then used it to generated mixed nanoparticles with poly(lactic-co-glycolic acid) (PLGA). PLGA has been approved by the US Food and Drug Administration (FDA) for certain human applications, and its various advantages of non-toxicity, biodegradability, biocompatibility, and controlled-/sustained-release efficacy have been proven. Due to the integrated properties of this novel system, namely tumor penetration, nuclear targeting, and pH-responsive features, we hypothesized that this system could be promising in the co-delivery of CUR and pSUR, and be effective in the treatment of cancers (Figure 1).

## Materials and Methods

### Materials

PLGA (LA/GA = 75:25; MW = 15 Kd) was obtained from the Jinan Daigang Biomaterial Co., Ltd. (Jinan, China). CBZ-L-HIS(DNP)-OH was purchased from the Jill Biochemical Co., Ltd. (Shanghai, China). [W5R4]K was received from the Phtdpeptides Pharmaceutical Co., Ltd (Zhengzhou, China). Survivin shRNA plasmid (pSUR) was constructed by the TsingKe Biological Technology Co., Ltd. (Chengdu, China). Curcumin (CUR), polyethylene glycol (PEG, MW =2 kD), polyvinyl alcohol (PVA, MW = 30–70 Kd, HD: 80%), Hochest 33258, poly-L-lysine (PLL, MW = 15–30 Kd), and 3-(4,5-dimethylthiazol-2-yl)-2,5-diphenyltetrazoliumbromide (MTT) were procured from the Sigma-Aldrich Chemical Co. (St. Louis, MO, USA). All other reagents were of analytical grade and were used without further purification. Anti-survivin and anti-cleaved caspase-3 antibodies were produced from the Abcam Trading (Shanghai) Co., Ltd. (China).

### Cell Culture

Human embryonic kidney 293 (HEK293), SKOV-3, and Hela cells were all obtained from the American Type Culture Collection (ATCC). All cells were cultured in an incubator with a humidified atmosphere containing 5% CO_2_ at 37°C and were maintained in DMEM supplemented with 10% fetal bovine serum.

### Anti-tumor Effect of CUR and pSUR *in vitro*

#### Cell Viability Assay by MTT

SKOV-3 and Hela cells were seeded and cultured in 96-well plates and transfected with pRNA (0.1 μg) using Lipofectamine® 3,000 for 24 h. Then, the medium was replaced by fresh medium containing various concentrations (2.5, 5, 10, and 20 μg/mL) CUR. After 48 h, MTT solution (20 μL, 5 mg/mL) was added, and the cells continued to be incubated for 4 h. The formazan crystals formed by the living cells were dissolved in DMSO (150 μL). The absorbances at 570 nm were read using a Spectra MAX M5 microplate spectrophotometer (Molecular Devices), and the inhibition rates were calculated.

#### RT-qPCR

SKOV-3 and Hela cells were seeded and cultured in 6-well plates and then transfected with pSUR (2.5 μg) using Lipofectamine® 3000. After 4 h, the transfection mixture was replaced by a fresh medium containing CUR (10 μg/mL), and cells continued to be cultured for another 24 h. Total RNA was extracted from the treated SKOV-3 and Hela cells using a TRIZOL reagent (Hung et al., 2012). After being purified through a genomic DNA elimination reaction, the extracted RNA was subsequently reversed-transcribed into cDNA. Real-time RT-PCR was performed using the IQ™ SYBR Green PCR Supermix PCR kit (BIO-RAD, USA) with the Bio-Rad iCycler apparatus system. The reaction conditions were: 95°C for 3 min, 95°C for 15 s, and 60°C for 1 min (44 cycles). The pair of primers for target genes are designed as: Survivin (forward, 5′-AGATGACGACCCCATAGAGG-3′; reverse, 5′-ATTGTTGGTTTCCTTTGCAATTTTG-3′); GAPDH (forward, 5′-GCACCGTCAAGGCTGAGAAC-3′; reverse, 5′-TGGTGAAGACGCCAGTGGA-3′).

#### Western Blot Analysis

SKOV-3 and Hela cells were cultured and treated as described in section Syntheses of [W5R4]K-PEG2K-PHIS. Total proteins were harvested and analyzed by western blot. The protein was separated on a SDS-PAGE, transferred onto a PVDF membrane, and blocked and incubated with the primary antibody at 4°C overnight. The proteins were detected by incubating membranes with a horseradish peroxidase (HRP)-conjugated secondary antibody. The binds were visualized using an enhanced chemiluminescence detection kit (Pierce, Rockford, IL, USA). The membranes were re-probed with a β-actin antibody to confirm equal protein loading.

### Polymer Synthesis

#### Syntheses of PEG_2K_-PHIS

N^α^-CBZ-N^im^-DNP-L-histidine was first dissolved in anhydrous THF followed by the addition of thionyl chloride under stirring. The reaction was performing for 12 h at room temperature. Then an excess of anhydrous diethyl ether was added to obtain the crystals of N^im^-DNP-L-histidine carboxyanhydride hydrochloride (N^im^-DNP-L-histidine NCA^.^HCl). The powder was subsequently washed, filtered, and dried under vacuum. Next, poly(N^im^-DNP-L-histidine) [PHIS(DNP)] was synthesized by ring-opening polymerization (ROP) of protected N^im^-DNP-L-histidine NCA^.^HCl with a predetermined amount of isopropylamine initiator, to obtain different polymerization degrees and molecular weights (Lee et al., 2003).

Meanwhile, COOH-PEG_2K_-COOH (CPC) was synthesized according to our previous paper (Yu et al., 2016). Briefly, PEG_2K_, succinic anhydride, and 4-dimethylaminopyridine (DMAP) were dissolved in dichloromethane (DCM) solution and stirred at room temperature (RT) over 24 h. Thereafter the solvent was evaporated, and the crude product CPC was purified by the recrystallization process from isopropyl alcohol. CPC was collected via vacuum filtration.

Finally, CPC was hydrophobically modified by chemical conjugation of PHIS(DNP) through amide formation to obtain a PEG_2K_-PHIS(DNP) diblock copolymer. Briefly, CPC was dissolved in anhydrous N,N-dimethylformamide (DMF). Then, EDCI, NHS, and PHIS(DNP) were added, respectively, under stirring. After reacting for 72 h, the solution was dialyzed (MWCO 3,500 Da) to remove small fragments. The product PEG_2K_-PHIS(DNP) was obtained by freeze-drying, and was then deprotected to yield PEG_2K_-PHIS (PH) by thiolysis with 2-mercaptoethanol. The chemical structure of the intermediates and PH were analyzed by using ^1^H-NMR.

#### Syntheses of [W5R4]K-PEG_2K_-PHIS

The diblock copolymer PH was dissolved in anhydrous DMF, followed by the addition of EDCI and NHS under stirring. The mixture was continuously stirred for an additional 12 h under a nitrogen atmosphere, and then cycle peptide [W5R4]K dissolved in anhydrous DMF was added dropwise. The reaction was continued at room temperature for 72 h. The product [W5R4]K-PEG2K-PHIS (WPH) was purified by dialyzing (MWCO 3,500 Da), followed by lyophilization. The chemical structure of WPH was confirmed by ^1^H-NMR.

### Preparation of CUR/pSUR-NPs

CUR/pSUR-NPs were constructed by using the W/O/W double emulsification technique as reported previously (Xu et al., 2016a). Briefly, CUR, PLGA, and WPH were dissolved in DCM as the organic phase, and then emulsified with the internal aqueous phase (containing the pSUR gene) by sonication at 45 W for 20 s in an ice bath. PVA solution (1%, 4 mL) was added into the primary emulsion and emulsified by sonication (75 W for 2 min) to produce the multiple emulsion (W/O/W). PVA (1%, 2 mL) was further added and the final W/O/W emulsion was obtained by sonication at 40 W for 20 s. The organic solvent was immediately evaporated under vacuum at 37°C. The obtained colloidal solution was centrifuged (13,300 rpm, 40 min) at 4°C to remove PVA and the unencapsulated drug/gene. The precipitate was re-dispersed in PBS (pH 7.4) to get CUR/pSUR-NPs. This process was also used to prepare the contrast agents including the single drug or gene loaded CUR-NPs and pSUR-NPs.

### Characterization of CUR/pSUR-NPs

#### Entrapment Efficiency and Drug Loading

Entrapment efficiency (EE%) and drug loading capacity (DL%) of CUR/pSUR-NPs were calculated as previously described (Xu et al., 2016b; Feng et al., 2020). Firstly, the supernatant and sediment were collected after centrifuging the CUR/pSUR-NPs colloidal solution. Then the supernatant was incubated with Hoechst 33258 (0.15 μg/mL), followed by a measurement of the fluorescence intensity by a spectrometer (Perkin Elmer, USA) at a 358 nm excitation wavelength and a 457 nm emission wavelength, which was used to calculate the amount of pSUR gene unentrapped into NPs. Meanwhile, the sediment was dissolved in methanol, and the content of CUR entrapped into CUR/pSUR-NPs was measured by UV-vis spectrophotometer (UV-2550, Shimadzu, Japan) with the detection wavelength at 420 nm. EE% and DL% of pSUR and CUR were monitored by using the following formulae:

$$\begin{array}{l} \text{EE}\%(\text{pSUR})\\ \text{=}\left(\frac{\text{initial pSUR gene content content of pSUR gene unentrapped}}{\text{initial pSUR gene content}}\right)\times \text{100}\\ \text{DL}\%(\text{pSUR})\\ \text{=}\left(\frac{\text{initial pSUR gene content content of pSUR gene unentrapped}}{\text{weight of nanoparticles}}\right)\times \text{100}\\ \text{EE}\%(\text{CUR})\\ \text{=}\left(\frac{\text{content of CUR entrapped in nanoparticles}}{\text{initial CUR content}}\right)\times \text{100}\\ \text{DL}\%(\text{CUR})\\ \text{=}\left(\frac{\text{content of CUR entrapped in nanoparticles}}{\text{weight of nanoparticles}}\right)\times \text{100} \end{array}$$

#### Particle Size and Zeta Potential

The mean diameter and zeta-potential of CUR/pSUR-NPs were determined by a Zetasizer Nano ZS (Malvern Instruments, Ltd., Malvern, Worcestershire, U.K.) at 25°C. Additionally, to investigate the influence of pH, CUR/pSUR-NPs were re-dispersed in PBS pH 4.5 and PBS pH 7.4, respectively, and the particle size and zeta-potential were further measured after shaking at 37°C at a gentle rate of 100 rpm for 24 h. A total of 3 parallel runs were carried out for each measurement, and all data were expressed as the mean ± SD.

#### Transmission Electron Microscopy (TEM)

For morphological observations, a small drop of the CUR/pSUR-NPs dispersion was placed on a copper grid and then negatively stained with a 2% (w/v) phosphotungstic acid solution for 2 min. Excess fluid was removed using a piece of filter paper. Morphology was examined on a transmission electron microscope (H-600, Hitachi, Ltd., Japan).

#### Differential Scanning Calorimetry (DSC)

Differential scanning calorimetry (DSC, 200PC, Netzsch, Karlsruhe, Germany) was used to perform the physical property of CUR loaded in CUR/pSUR-NPs. Freeze-dried CUR/pSUR-NPs, free CUR, blank nanoparticle, and the physical mixture of the latter two with the same mass ratios as those in CUR/pSUR-NPs were heated from 20 to 400°C at a speed of 10°C/min.

#### *In vitro* CUR and pSUR Release

The release profiles of CUR and pSUR in CUR/pSUR-NPs were evaluated in phosphate buffer (PBS) containing 1 % (w/v) Tween 80 at pH 7.4 and 4.5, respectively. CUR/pSUR-NPs dispersed in PBS were divided into 20 parts and shaken at 37°C at a gentle rate of 100 rpm. One part was taken out at a definite time interval, and then treated with centrifugation. The amount of released pSUR in the supernatant and CUR remained in the sediment were investigated by the same methods as described at section Entrapment Efficiency and Drug Loading.

#### Hemolysis Assay

We first obtained 2% erythrocyte dispersion and then treated it with various amounts of formulations. Deionized water and normal saline (NS) were, respectively, employed as the positive and negative control. After incubation at 37°C for 3 h, all the samples were centrifuged. The absorbance (A) of the obtained supernatant was monitored by UV-Vis spectrophotometer (Perkin-Elmer Lambda35, USA) at 545 nm. The percentage of the samples-induced hemolysis was measured as follows:

$$\begin{array}{l} \text{Hemolysis }\left(\%\right)\\ \text{=}\frac{\text{A of sample }-\text{ A of negative control}}{\text{A of positive control }-\text{ A of negative control}}\times \text{100} \end{array}$$

### *In vitro* Experiments

#### Cellular Uptake of Nanoparticles

SKOV-3 and Hela cells were seeded and cultured in 24-well plates overnight. The original medium was replaced by a fresh medium containing CUR/pSUR-NPs (shRNA labeled with red fluorescence). After incubation for 4h, the supernatant was removed and the cells were washed twice with cold PBS (pH 7.4). Cells were fixed in 4 % paraformaldehyde for 10 min at room temperature, and nuclei were stained using Hoechst 33258 (2.5 μg/mL) for 15 min in the dark. Finally, cells were visualized and imaged using a fluorescence microscopy.

#### Lysosomal Escape and Nuclear Targeting

In order to investigate the lysosomal escape and nuclear targeting properties of nanocarriers, a fluorescent lipophilic dye DiI was incorporated into the nanoparticles by the same W/O/W method as described in the section Preparation of CUR/pSUR-NPs. After overnight culturing, the medium was removed and replaced by a fresh medium containing DiI-NPs. After a 4 h incubation, the cells were washed, and stained by LysoTracker Green probe (Life technologies, USA) and Hoechst 33258. After being fixed with 4% paraformaldehyde, finally, the subcellular localization was observed using a fluorescence microscope. Meanwhile, to investigate the role of the PHIS and [W5R4]K segments, different nanoparticles, namely PLGA-NPs (P-NPs) and PLGA/PH-NPs (P/PH-NPs) were used as the contrast.

#### Penetration in Multicellular Tumor Spheroids

SKOV-3 and Hela tumor spheroids *in vitro* were first prepared. Low melting point agarose (2%, w/v) was added into the flat bottom of a 96-well plate, and 3,000 cells were seeded on it. Cells were cultured until uniform spheroids formed, then DiI-NPs were added. After further incubation for 12 h, the spheroids were rinsed, and their fluorescence intensity was observed with a confocal microscopy (Zeiss LSM 800 Airyscan).

#### Transfection of Nanoparticles

Gene transfection efficiency was tested using pGFP as reporter gene in HEK293 cells. pGFP-NPs (namely pGFP-P/WPH-NPs here) were prepared by the W/O/W method as described in the section Preparation of CUR/pSUR-NPs. Cells were seeded into 24-well plates. After 24 h, the old media were replaced by a fresh medium with the pGFP-NPs (4 μg of pDNA per well). The NPs containing a medium was replaced with a fresh medium at 4 h. After 48 h incubation, cells were imaged under fluorescence microscopy. Meanwhile, the transfection efficiency of contrast agents were also investigated, including pGFP-P-NPs which were conducted by PLGA polymer only, and pGFP-P/PH-NPs which were conducted by PLGA and PEG-PHIS polymers.

To investigate the influence of serum components, transfection efficiency of pGFP-NPs were further tested in a serum-free and serum-contained medium. After incubating the cells for 24 h, the old media were replaced by a fresh serum-free or serum-contained medium with the pGFP-NPs (4 μg pDNA per well). This medium was replaced with a fresh serum-contained medium at 4 h. After 48 h, cells were imaged using fluorescence microscopy. After that, the cell lysates were harvested and the transfection efficiency was evaluated by a FACS Calibur flow cytometer (FACS Canto II, BD Biosciences, San Jose, CA). Lipofectamine® 2000 was used as control.

Transfection efficiencies of P/WPH-NPs on SKOV-3 and Hela cells were also investigated, according to the same procedure on HEK 293.

#### Cell Viability Assay by MTT

SKOV-3 and Hela cells were seeded and cultured in 96-well plates. The cells were incubated with CUR-NPs, pSUR-NPs, physical mixture of two formers (namely Mixture-NPs), CUR/pSUR-NPs, and free CUR for 24 h. The concentrations of pSUR were 0.5 and 1 μg/mL, and corresponding concentrations of CUR were 1 and 10 μg/mL, respectively. Subsequent experimental steps were the same as described in section Syntheses of PEG_2K_-PHIS.

#### Cell Apoptosis Analysis

The apoptosis of SKOV-3 and Hela cells was determined by an Annexin V-FITC/PI apoptosis detection kit. Briefly, after treatment with CUR, CUR-NPs, pSUR-NPs, Mixture-NPs, and CUR/pSUR-NPs at 37°C for 12 h, cells were trypsinized and collected by centrifugation. The cells were washed and resuspended in binding buffer. The cells were, respectively, stained with Annexin V-FITC and PI, followed by flow cytometry analysis (Becton-Dickinson, USA).

#### Immunofluorescence Staining of Cleaved Caspase-3

SKOV-3 and Hela cells were seeded and cultured in 24-well plates. CUR, CUR-NPs, pSUR-NPs, Mixture-NPs, and CUR/pSUR-NPs were incubated for 24 h. Cells were fixed with paraformaldehyde, permeabilized with 0.1% Triton X-100, blocked with bovine serum albumin (BSA), and incubated with a cleaved caspase-3 antibody. Next, the immunocomplexes were incubated with a Alexa 488 anti-mouse secondary antibody and stained by Hoechst 33258. The signals and colocalization were assessed by fluorescence microscopy. The fluorescence intensity was measured by Image J software.

#### Nuclear Morphological Analysis

Apoptotic morphological changes were evaluated using Hoechst 33258 staining. After 48 h of CUR, CUR-NPs, pSUR-NPs, Mixture-NPs, and CUR/pSUR-NPs incubation, the cells were fixed with paraformaldehyde and stained with Hoechst 33258. Then the nuclear morphology of cells was observed by fluorescence microscopy.

### Statistical Analysis

All quantitative data were presented as mean ± standard deviation (SD). Statistical comparisons between two groups were analyzed using an unpaired student *t*-test, and comparisons between multiple groups were assessed by one-way ANOVA. A value of *P* < 0.05 was considered significant and *P* < 0.01 was considered highly significant.

## Results and Discussion

### Anti-tumor Effects of Free CUR and pSUR *in vitro*

**Figure 2:**
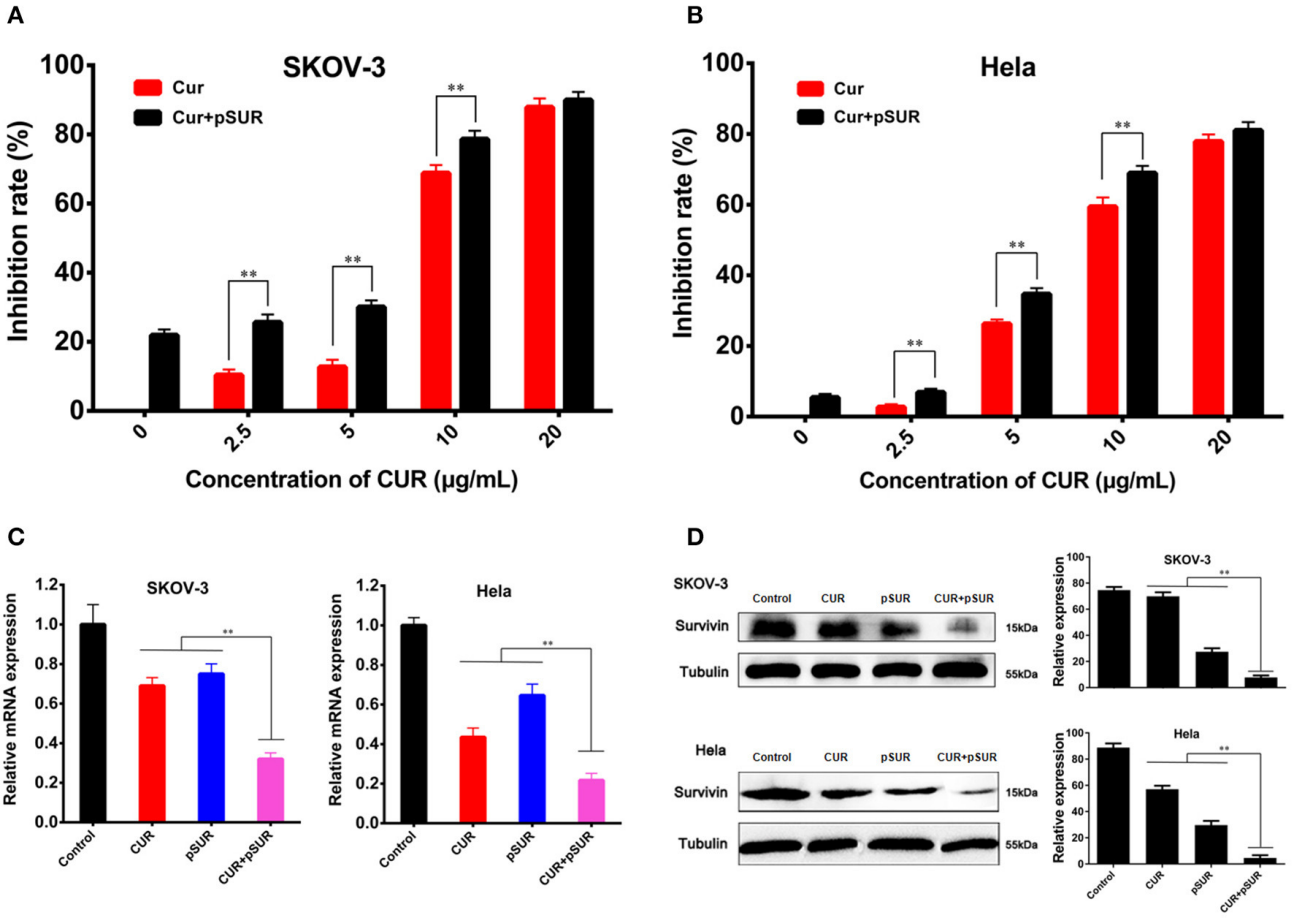
Combined treatment of CUR and pSUR enhanced anti-tumor effects *in vitro*. Growth inhibition of SKOV-3 **(A)** and Hela **(B)** cells after treatment with varying concentrations of CUR or the combination of CUR and pSUR. Relative survivin mRNA **(C)** and protein **(D)** expression determined by RT-PCR and western blotting on SKOV-3 and Hela cells after treatment with CUR, pSUR, and their combination. **P < 0.01.

The inhibitory effects on the proliferation of pSUR combined with various concentrations of free CUR were primarily evaluated by the MTT assay in SKOV-3 and Hela tumor cell lines. As shown in Figures 2A,B, both cells lines were inhibited with varying concentrations (2.5–20 μg/mL) of CUR. Additionally, the combined therapy displayed concentration-dependent cell growth inhibition activities. Notably, co-administration significantly elevated the inhibitory effects compared with the single use of CUR, revealing the synergistic effect arising from the co-delivery of CUR and pSUR.

The suppression of survivin expression was further evaluated using RT-PCR and western blot (Figures 2C,D). pSUR showed significant downregulation effects of the survivin mRNA/protein on both SKOV-3 and Hela cell lines, confirming that pSUR conducted in this work was indeed able to knock down the survivin expression. CUR also showed a strong inhibitory effect. The results were in agreement with the earlier studies, in which most of the cell types displayed a decreased expression of survivin following curcumin treatment (Watson et al., 2010; Khaw et al., 2013). More importantly, pSUR and CUR co-administration exhibited the strongest inhibition, ascribed to the synergistic effect of the gene and drug.

### Synthesis and Characterization of Polymer Conjugates

**Figure 3:**
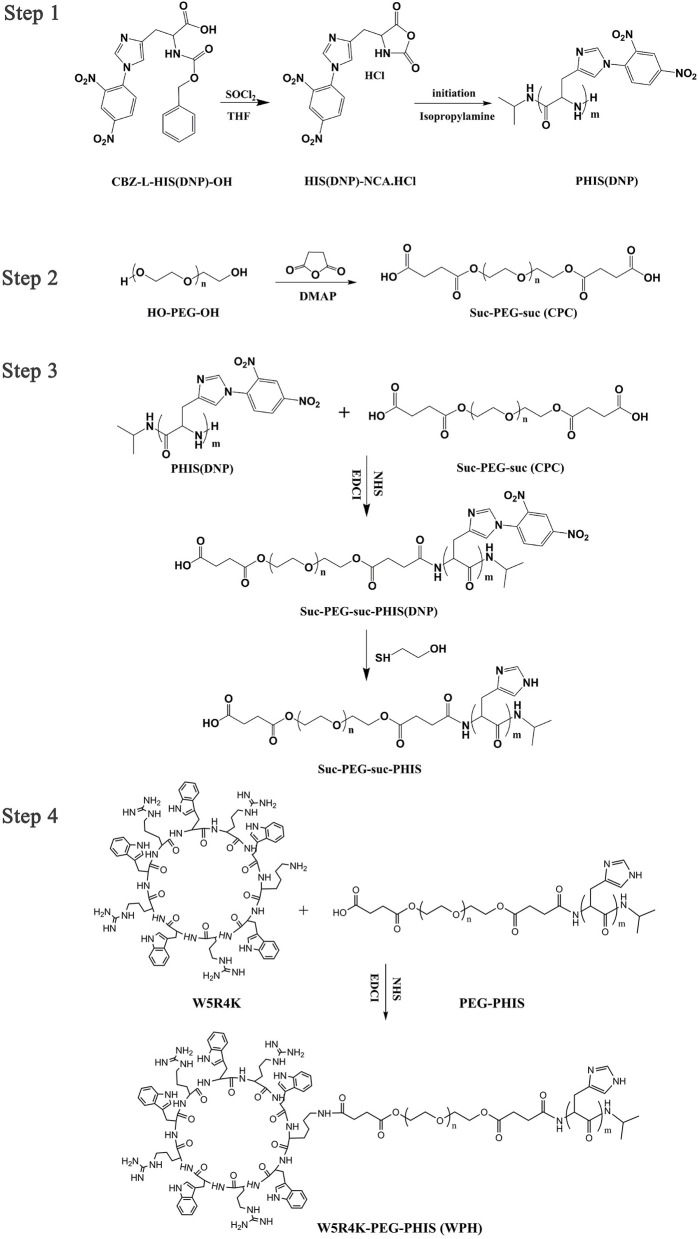
Overall scheme for the synthesis of WPH.

**Figure 4:**
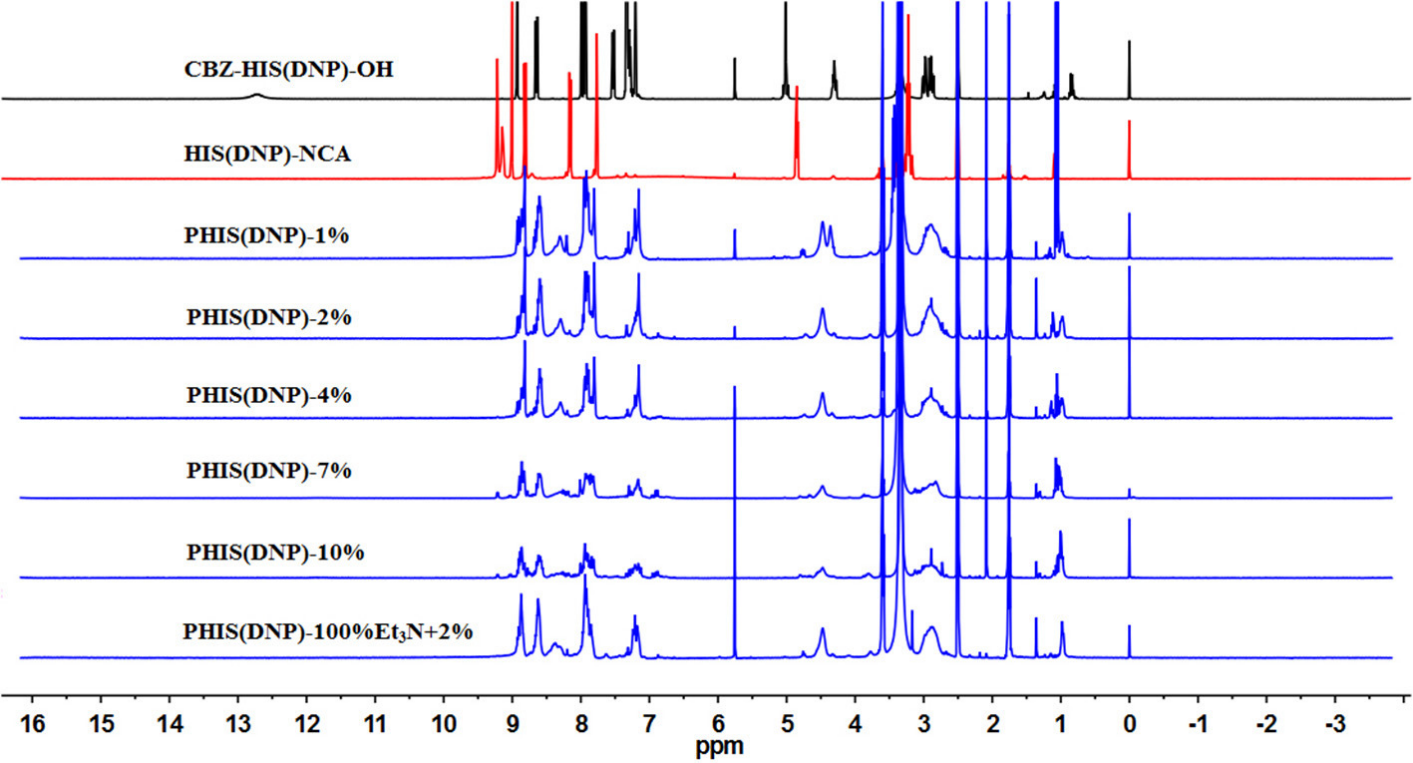
^1^HNMR spectrum of PHIS(DNP) with different polymerization degrees (P.D.). The P.D. of polyhistidine was calculated by comparing the integrations of hydrogen protons on isopropylamine methyl and hydrogen protons of the DNP group on PHIS(DNP).

An overall synthetic route for preparation of [W5R4]K-PEG_2K_-PHIS (WPH) was present in Figure 3. The structure of N^im^-DNP-L-histidine NCA^.^HCl was confirmed by ^1^H NMR [400 MHz, d6-DMSO trimethylsilyl (TMS)]: 8.25–9.26 (2,4-dinitrophenyl protons, DNP), 8.80 (-NH-), 8.25 (-N-CH=C-), 7.84 (-N=CH-), 4.88 (-CH-), and 3.25 (-CH_2_-) (Figure 4). The ring opening polymerization of N^im^-DNP-L-histidine NCA^.^HCl was then performed using different molar ratios of the initiator to monomer (I/M ratio). The polymerization degree (P.D.) of polyhistidine was calculated by comparing the integrations of hydrogen protons on isopropylamine methyl and hydrogen protons of DNP group on PHIS(DNP) (Table 1). ^1^H NMR [400 MHz, d6-DMSO trimethylsilyl (TMS)] for PHIS(DNP) with various P.D.: 8.21–9.00 (2,4-dinitrophenyl protons, DNP), 7.98 (-N-CH=C-), 7.26 (-N=CH-), 4.41 (-CH-), 3.35 (-CH_2_-), and 1.00 (-(CH_3_)_2_) (Figure 4). Results showed that P.D. of PHIS(DNP) decreased with the increasing I/M ratios from 1 to 7%, which was consistent with the previous study (Lee et al., 2003). Although when the I/M ratios further increased to 10%, P.D. no longer changed. The high I/M ratios of 7 and 10% produced rather low-molecular-weight polymers, likely due to the excessive initiators and faster propagation rates. Additionally, the P.D. of PHIS(DNP) initiated by (100% triethylamine + 2% isopropylamine) was lower than that by 2% isopropylamine only. This observation is not well-understood, but could be related to the incomplete neutralization of hydrochloride from N^im^-DNP-L-histidine NCA^.^HCl. The residual salts might influence the following initiation by isopropylamine.

**Table 1:** Polymerization degrees (P.D.) of PHIS(DNP) with various I/M molar radios.

**Initiator with different I/M molar radios**	**P.D. of PHIS(DNP)**
1% isopropylamine	22–24
2% isopropylamine	18–20
4% isopropylamine	17–18
7% isopropylamine	5–6
10% isopropylamine	5–6
100% triethylamine + 2% isopropylamine	12–14

**Figure 5:**
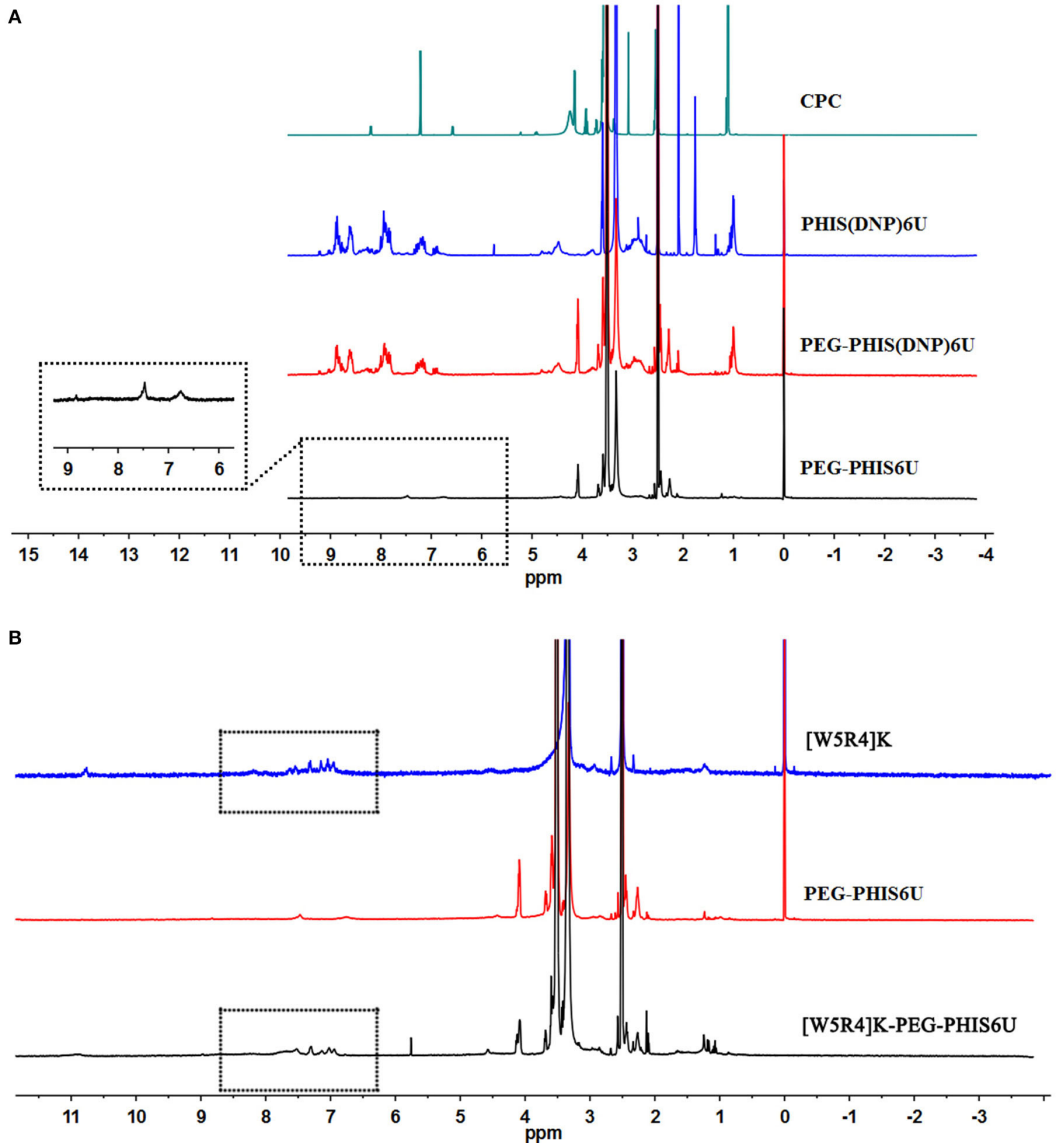
^1^H-NMR spectrum of CPC, PEG-PHIS(DNP)_6U_, PEG-PHIS_6U_, and WPH_6U_. **(A)** PEG-PHIS_6U_ was successfully synthesized by the removal of the benzyloxylcarbonyl group. **(B)** WPH_6U_ was successfully synthesized for containing the principal proton peaks related to the [W5R4]K, PEG, and PHIS moieties.

**Figure 6:**
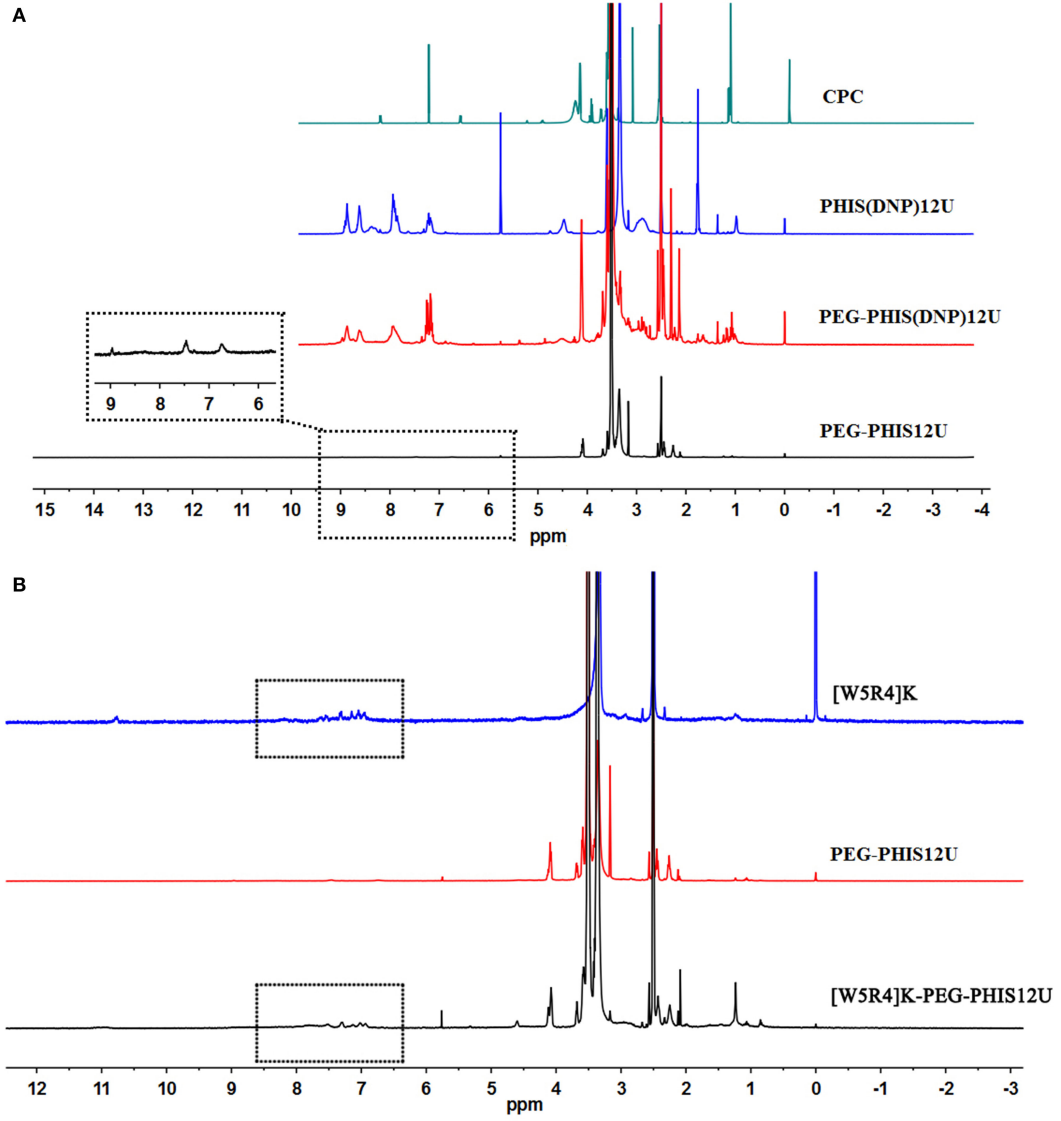
^1^H-NMR spectrum of CPC, PEG-PHIS(DNP)_12U_, PEG-PHIS_12U_, and WPH_12U_. **(A)** PEG-PHIS_12U_ was successfully synthesized by the removal of the benzyloxylcarbonyl group. **(B)** WPH_12U_ was successfully synthesized for containing the principal proton peaks related to the [W5R4]K, PEG, and PHIS moieties.

**Figure 7:**
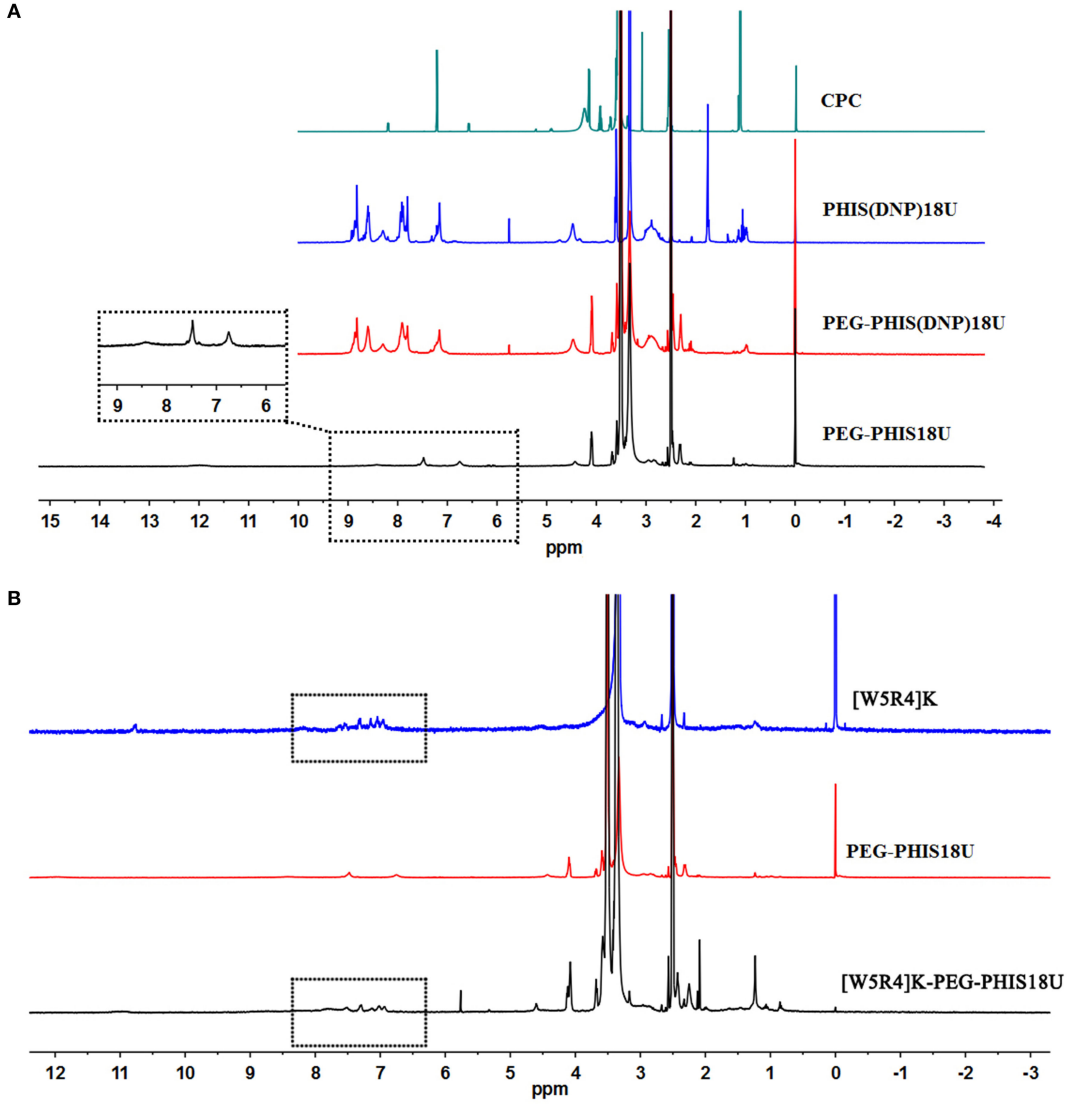
^1^H-NMR spectrum of CPC, PEG-PHIS(DNP)_18U_, PEG-PHIS_18U_, and WPH_18U_. **(A)** PEG-PHIS_18U_ was successfully synthesized by the removal of the benzyloxylcarbonyl group. **(B)** WPH_18U_ was successfully synthesized for containing the principal proton peaks related to the [W5R4]K, PEG, and PHIS moieties.

PHIS(DNP) with P.D. of 6, 12, and 18, namely PHIS(DNP)_6U_, PHIS(DNP)_12U_, and PHIS(DNP)_18U_ were then selected to prepare PEG-PHIS(DNP)_6U_, PEG-PHIS(DNP)_12U_, and PEG-PHIS(DNP)_18U_, respectively. The structures of these DNP-protected diblock polymers were confirmed by ^1^H NMR [400 MHz, d6-DMSO trimethylsilyl (TMS)]: 7.93–8.88 (2, 4-dinitrophenyl protons, DNP), 7.64 (-N-CH=C-), 3.65 (-CH_2_CH_2_- in PEG group), 3.35 (-CH_2_- in PHIS group), 2.65–2.68 (-COCH_2_CH_2_CO-), and 1.00 (-(CH_3_)_2_) (Figures 5–7). Otherwise, removal of the benzyloxylcarbonyl group was proven by the disappearance of the peak of 7.93–8.88 (DNP). ^1^H NMR [400 MHz, d6-DMSO trimethylsilyl (TMS)] for PEG-PHIS (PH) with different P.D.: 8.21 (-N=CH-), 7.54 (-NH-C=O), 6.75 (-N-CH=C-), 3.65 (-CH_2_CH_2_- in PEG group), 3.35 (-CH_2_- in PHIS group), 2.65–2.68 (-COCH_2_CH_2_CO-), and 1.00 (-(CH_3_)_2_) (Figures 5–7). Interestingly, peaks of 6.8 and 7.5 were observed, indicating that an active carboxylic terminal was provided in PH, which could be used to synthesize WPH in the next reaction step.

Finally, the purified PH diblock copolymers were coupled with [W5R4]K to yield triblock copolymers with different P.D. of PHIS. ^1^H NMR [400 MHz, d6-DMSO trimethylsilyl (TMS)] for WPH: 6.96–8.22 (tryptophan indole ring proton), 3.65 (-CH_2_CH_2_- in PEG group), 3.35 (-CH_2_- in PHIS group), 3.30 (cyclic peptide proton), 2.65–2.68 (-COCH_2_CH_2_CO-), and 1.00 (-(CH_3_)_2_). The principal peaks related to the [W5R4]K, PEG, and PHIS moieties in the ^1^H-NMR spectrum of the final product indicated the successful synthesis of WPH (Figures 5–7).

### Preparation and Characterization of CUR/pSUR-NPs

**Figure 8:**
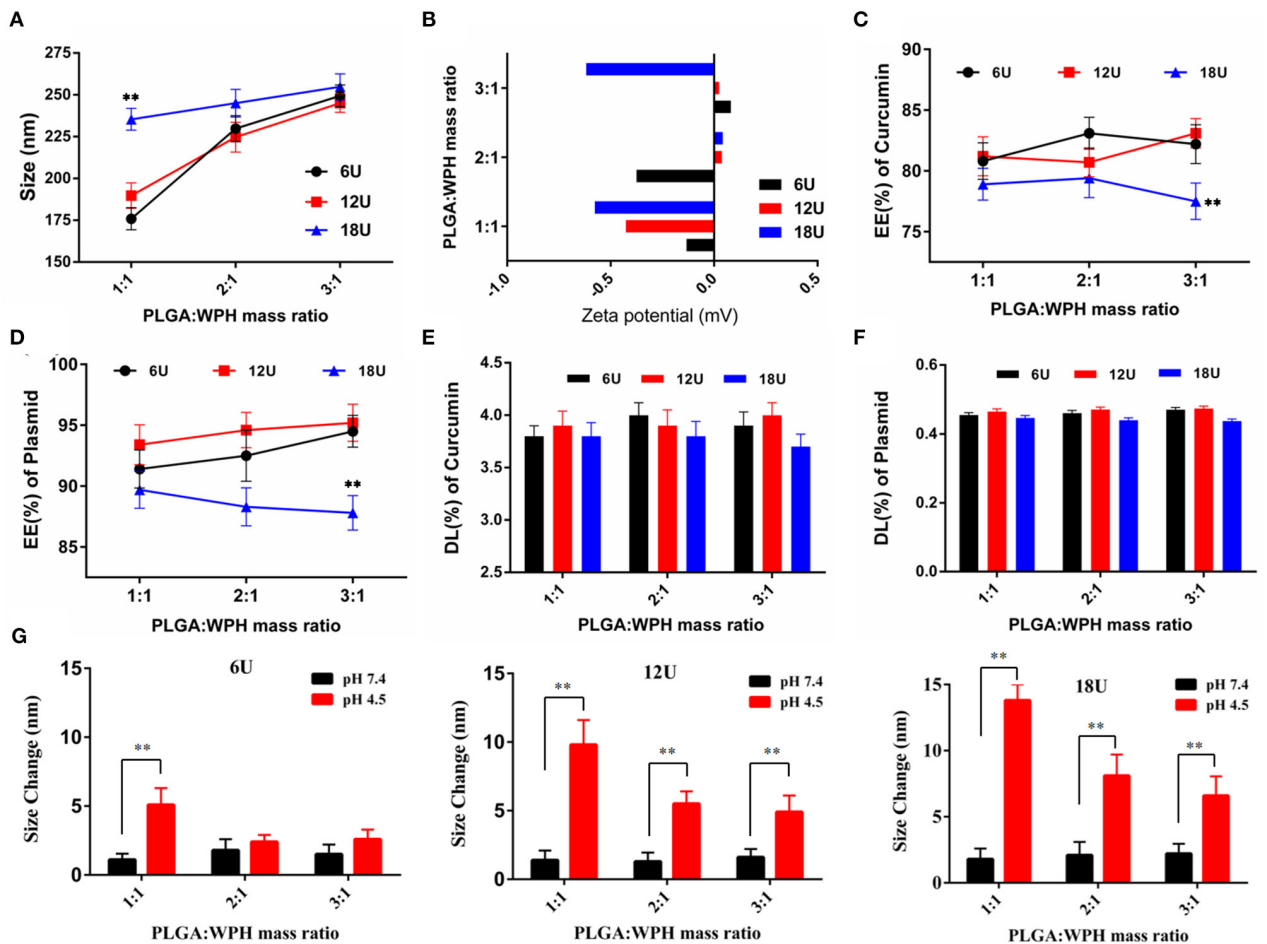
The effects of mass ratios of PLGA/WPH and P.D. of HIS in WPH triblock copolymer on diameter **(A)**, zeta potential **(B)**, EE% **(C,D)**, and DL% **(E,F)** of CUR and pSUR of CUR/pSUR-NPs. **(G)** Size changes of P/WPH-NPs in PBS pH 7.4 and pH 4.5 with different mass ratios of PLGA/WPH and P.D. of HIS. **P < 0.01.

The preparation of the CUR/pSUR-NPs was depicted in Figure 1. The mass ratios of PLGA/WPH and the different P.D. of HIS in WPH triblock copolymer that might affect the particle size, EE%, and DL% were screened. As shown in Figure 8, all CUR/pSUR-NPs exhibited good size distribution and high EE% and DL% of both CUR and pSUR, indicating NPs had good properties for co-loading chemical drugs and genes. All NPs were characterized with nearly neutral surface potential. As the PLGA/WPH mass ratio elevated from 1/1 to 3/1, the particle sizes significantly increased, while the EE and DL showed no remarkable significant change. The increased particle size was likely due to the increased oil droplets in the emulsifying process with the increased PLGA concentration (Hou et al., 2017). Otherwise, as the P.D. of HIS increased from 6 to 12, the particle sizes, EE and DL did not change significantly. Whereas, when the units further elevated to 18, the particle sizes markedly increased along with downward trends of EE and DL. The phenomenon was probably caused by the longer hydrophobic chain segment of PHIS, which could make the structure of NPs unstable during the solvent evaporation process. The pH-sensitive property of CUR/pSUR-NPs was further investigated. No obvious size changes were observed in pH 7.4, indicating the good stability of NPs in a normal physiological environment. However, in pH 4.5, the hydrated particle sizes were significantly increased, especially when the P.D. of HIS increased from 6 to 18, and the PLGA/WPH mass ratio was decreased from 3/1 to 1/1. This phenomenon might be related to the protonated PHIS blocks, which were positively charged in the acidic environment and caused the swelling of the core (Sun et al., 2015). The zeta potential was still neutral (data not shown), probably due to the PEG coating. Taking the parameters of sizes, EE, and DL into consideration, the optimal formulation with 1/1 PLGA/WPH mass ratio and 12 P.D. of HIS was selected and then its pharmaceutical properties were investigated.

**Figure 9:**
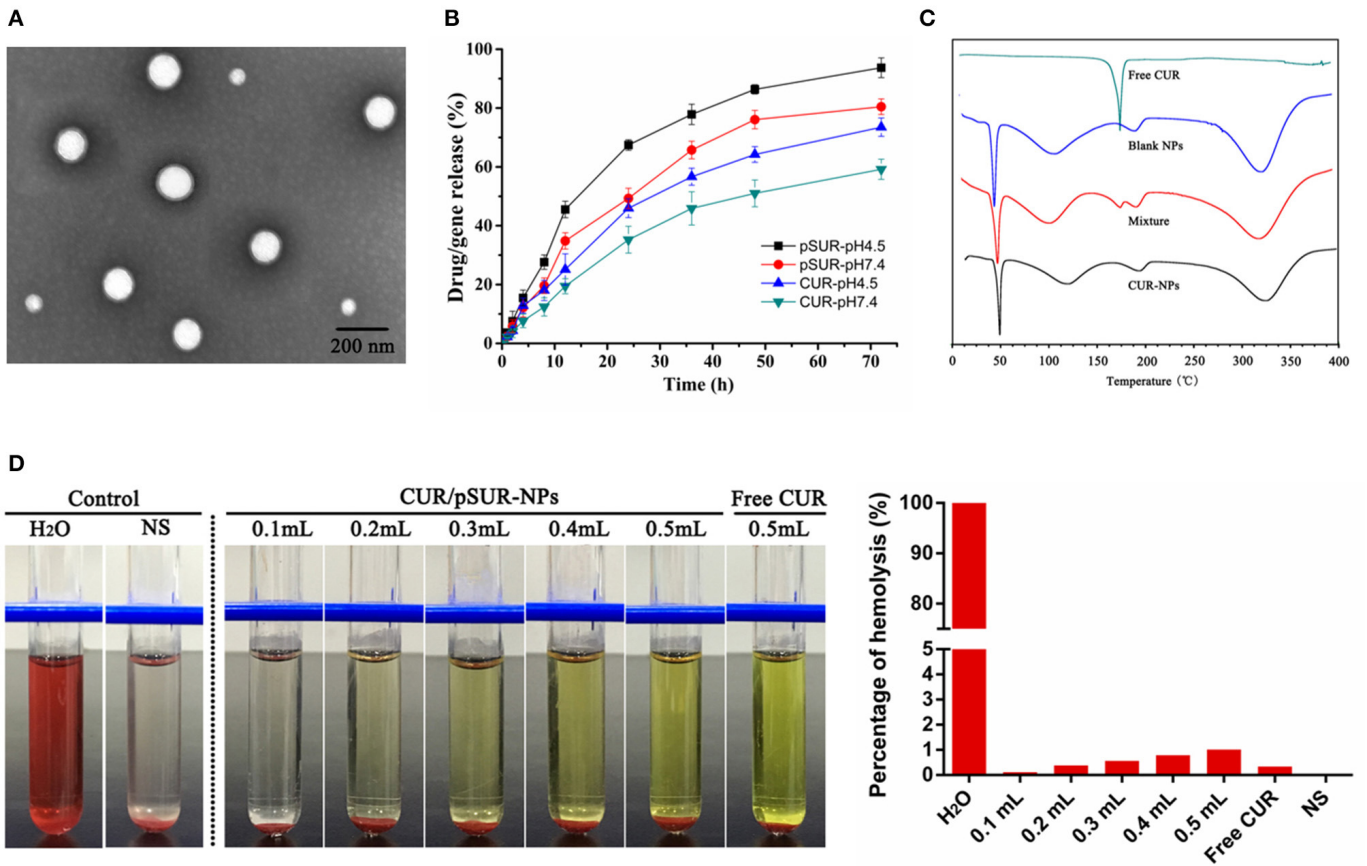
Pharmaceutical properties of CUR/pSUR-NPs. **(A)** Representative TEM image of CUR/pSUR-NPs. **(B)** *In vitro* drug release profiles of CUR and pSUR from CUR/pSUR-NPs at pH 4.5 and pH 7.4. **(C)** DSC curves of free CUR, Blank NPs, physical mixture of CUR and Blank NPs, and CUR/pSUR-NPs. **(D)** Images of hemolysis and the percentages of hemolysis after treatment with various volumes of CUR/pSUR-NPs.

The optimal NPs were uniformly distributed (PDI < 0.3, data not shown) with particles size around 180 nm. Otherwise, they had a good capacity for drug and gene incorporation with an EE% of 81.22 ± 1.56% and 93.41 ± 1.64%, and a DL% of 3.93 ± 0.12% and 0.46 ± 0.01%, respectively. TEM results certified that CUR/pSUR-NPs were homogeneous and spherical, being in good agreement with the narrow particle size distribution (Figure 9A).

*In vitro* release profiles of CUR and pSUR from CUR/pSUR-NPs were presented in Figure 9B. The CUR and pSUR loaded in NPs were progressively released in both of the release media with no obvious burst release. Moreover, typical pH-dependent releasing properties of CUR and pSUR were observed. At pH 7.4, 59.2 and 80.5% of CUR and pSUR were released at 72 h, while 93.7 and 73.5% were released at pH 4.5. The increased cumulative release might be caused by the protonated PHIS blocks in the acidic environment, that led to the swelling of core due to the same positive electrical charge (Sun et al., 2015). These release profiles can minimize the premature drug release during prolonged systemic circulation. In contrast, they can specifically enhance intracellular drug release, being beneficial to effective cancer treatment (Johnson et al., 2015). The pSUR gene displayed faster release rates than CUR. One reason might be that high hydrophilicity of the pSUR gene caused a relatively weaker interaction with hydrophobic polymer molecules than CUR. The observation was similar to our previous study (Xu et al., 2016b).

The DSC thermograms are shown in Figure 9C. An endothermic peak at 175.0°C was observed in the curve of free CUR powder. However, in the CUR/pSUR-NP group, the peak disappeared, and only two endothermic peaks at 118.2 and 193.6°C were presented, which corresponded to the PLGA and PVA's glass transition temperatures (Tg), respectively. Expectedly, the physical mixture presented all the three endothermic peaks, corresponding to the free CUR and the Tg of the PLGA and PVA. These results revealed that CUR had been successfully encapsulated into CUR/pSUR-NPs.

A hemolysis assay was conducted to evaluate CUR/pSUR-NPs' blood compatibility. All NPs treated groups had no visible hemolytic effect (Figure 9D). The percentage of erythrocyte hemolysis by all formulations was below 5%. The results suggested that CUR/pSUR-NPs had good blood compatibility and would be safe for i.v. injection.

### Cellular Uptake and Intracellular Distribution of P/WPH-NPs

**Figure 10:**
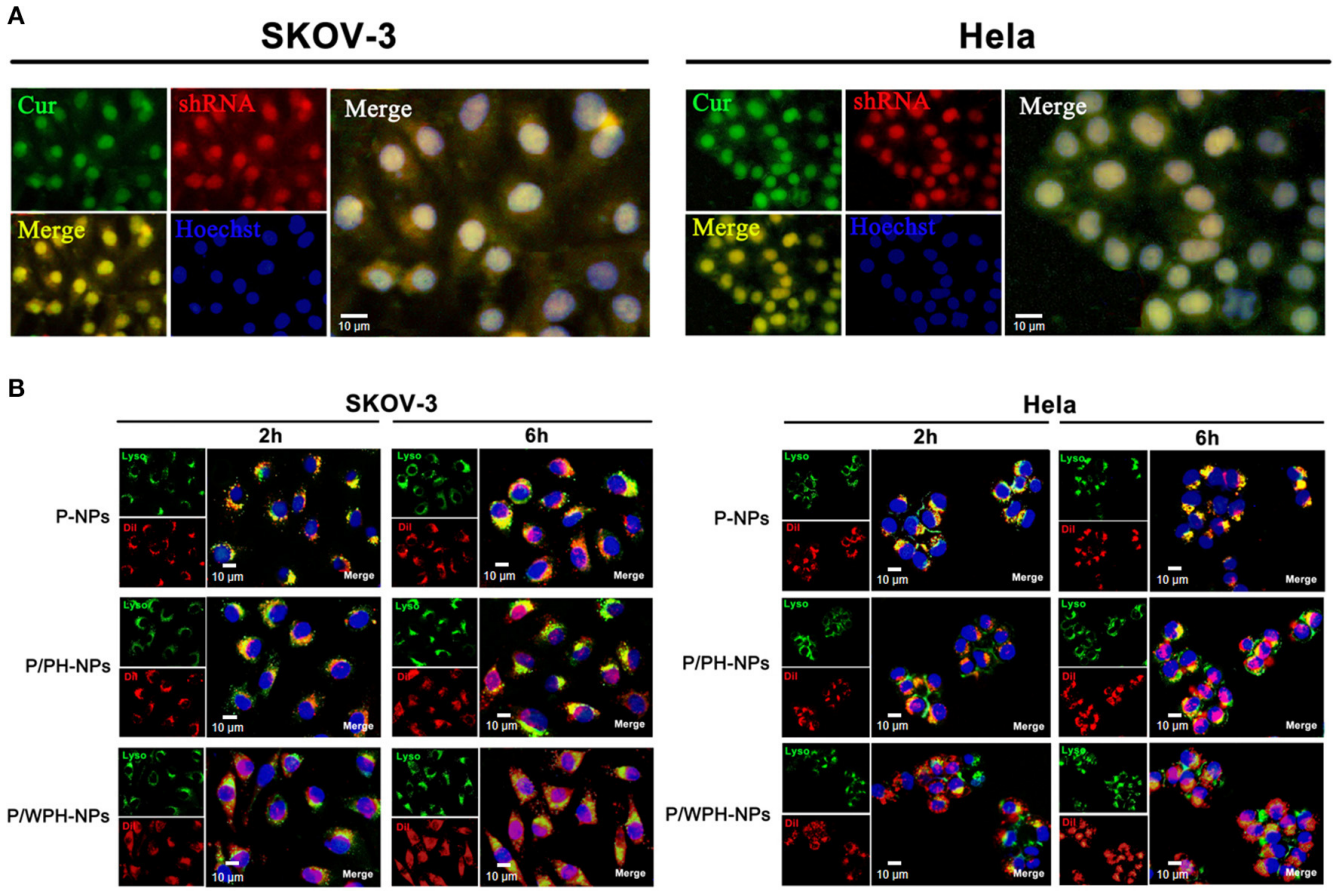
Cellular uptake and intracellular distribution of P/WPH-NPs (with 3 replicates). **(A)** Simultaneous cellular uptake of CUR and red fluorescence labeled pSUR on SKOV-3 and Hela tumor cells. The green, red, and blue colors represent the CUR, pSUR, and the nucleus, respectively. **(B)** Endosomal disruption and intracellular nuclear targeting of P/WPH-NPs on SKOV-3 and Hela cells. DiI (red fluorescence dye) was encapsulated in various nanocarriers. Endosome (Green) stained by Lysotracker® Green.

CUR (green autofluorescence) and red fluorescence labeled pSUR were used to investigate the co-uptake of the drug and plasmid in SKOV-3 and Hela cells. As Figure 10A showed, the green, red, and blue colors represent the CUR, pSUR, and the nucleus, respectively. The results showed that CUR and pSUR could be up-taken by the tumor cells simultaneously. Transporting the drug with the gene in the same carrier would be more advantageous for that they could be delivered to the same cells for combined actions and synergistic effects (Wang et al., 2006). Moreover, a great majority of CUR/pSUR-NPs subsequently targeted the nucleus, suggesting this nanovesicle is an excellent carrier that is suitable for the nuclear targeted delivery. The property might be related to the nuclear targeting profile of the [W5R4]K cell-penetrating peptide.

Additionally, the endosomal disruption of NPs in cells was further tested. To avoid an overlapped fluorescence signal between CUR and the Lysotracker (Green), we conducted DiI encapsulated PLGA/WPH-NPs (P/WPH-NPs) instead of CUR/pSUR-NPs. PLGA-NPs (P-NPs) and PLGA/PH-NPs (P/PH-NPs) label by DiI were used as the contrast. At 2 h, scattered green fluorescence inside of the cells was observed in the DiI-P/WPH-NPs and DiI-P/PH-NPs groups, indicating quick endosomal escape of the nanoparticles (Figure 10B). This phenomenon was probably attributed to the protonation of PHIS in the acidic tumor cells (Zhang et al., 2014). At 6 h, compared with the P-NPs and P/PH-NPs groups, a stronger superposition were seen as a pink color, implying lots of nanoparticles had escaped the endosome and entered the nucleus. The results confirmed our hypothesis of [W5R4]K for its potential of intracellular nuclear targeting delivery when utilized in drug/gene delivery polymeric nanosystems.

### Enhanced Penetration in Multicellular Tumor Spheroids

**Figure 11:**
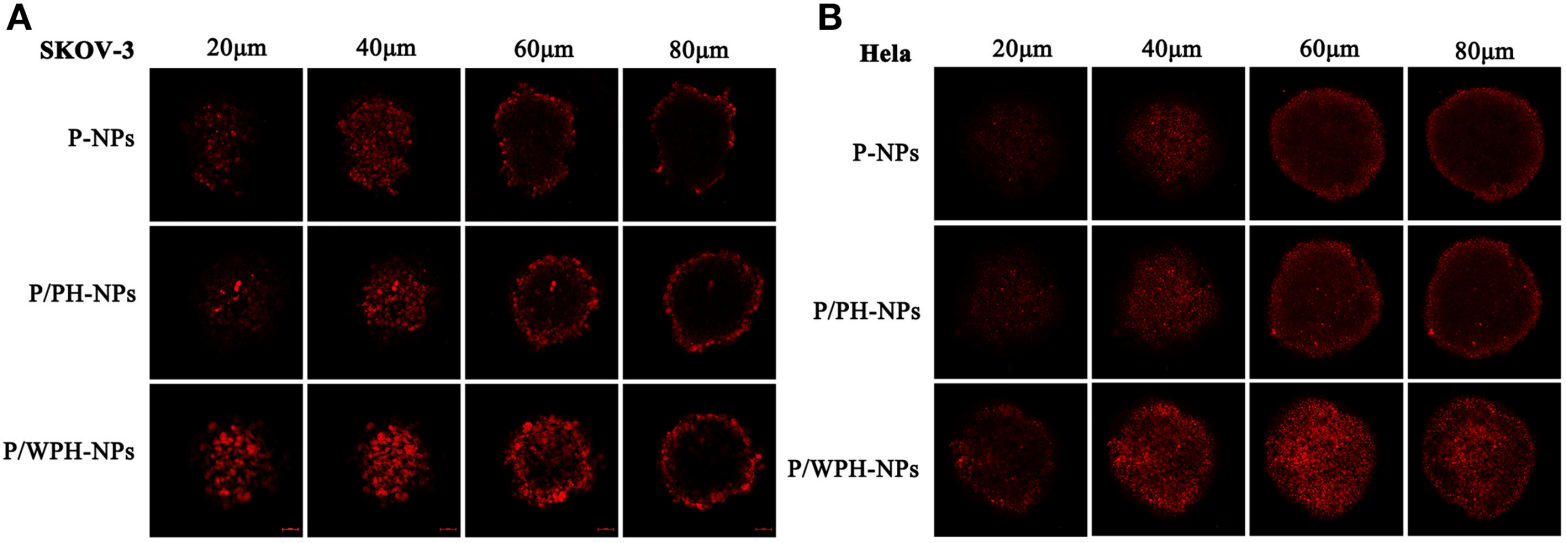
Confocal images of the uptake of DiI-labeled P-NPs, P/PH-NPs, and P/WPH-NPs on SKOV-3 **(A)** and Hela **(B)** tumor spheroids with different depths.

To obtain initial evidence of the nanoparticle tissue-penetrating ability, three-dimensional tumor spheroids were used (Wang et al., 2017). *In vitro* tumor spheroids could imitate the *in vivo* status of solid tumors, due to the characterizations of altered enzyme activity, poor drug penetration, and a viable rim with gradients of oxygen tension (Ruan et al., 2015). The distribution of various formulations in superficial sections of both the SKOV-3 and Hela tumor spheroid was observed (Figure 11). The fluorescent intensity of DiI encapsulated P-NPs and P/PH-NPs decreased dramatically along with the increase of section depths. The two-kind particles were difficult in penetrating into the deep region of the tumor spheroid probably due to their large size. Nevertheless, DiI-P/WPH-NPs exhibited the strongest internalization within the spheroids, indicating that [W5R4]K conjugation could enhance the nanomedicines' uptake in tumor spheroids and improve their drug delivery efficiency. This property is highly promising for increased NPs' accumulation in tumors interstitium after extravasation from the blood vessels.

### Transfection of P/WPH-NPs

**Figure 12:**
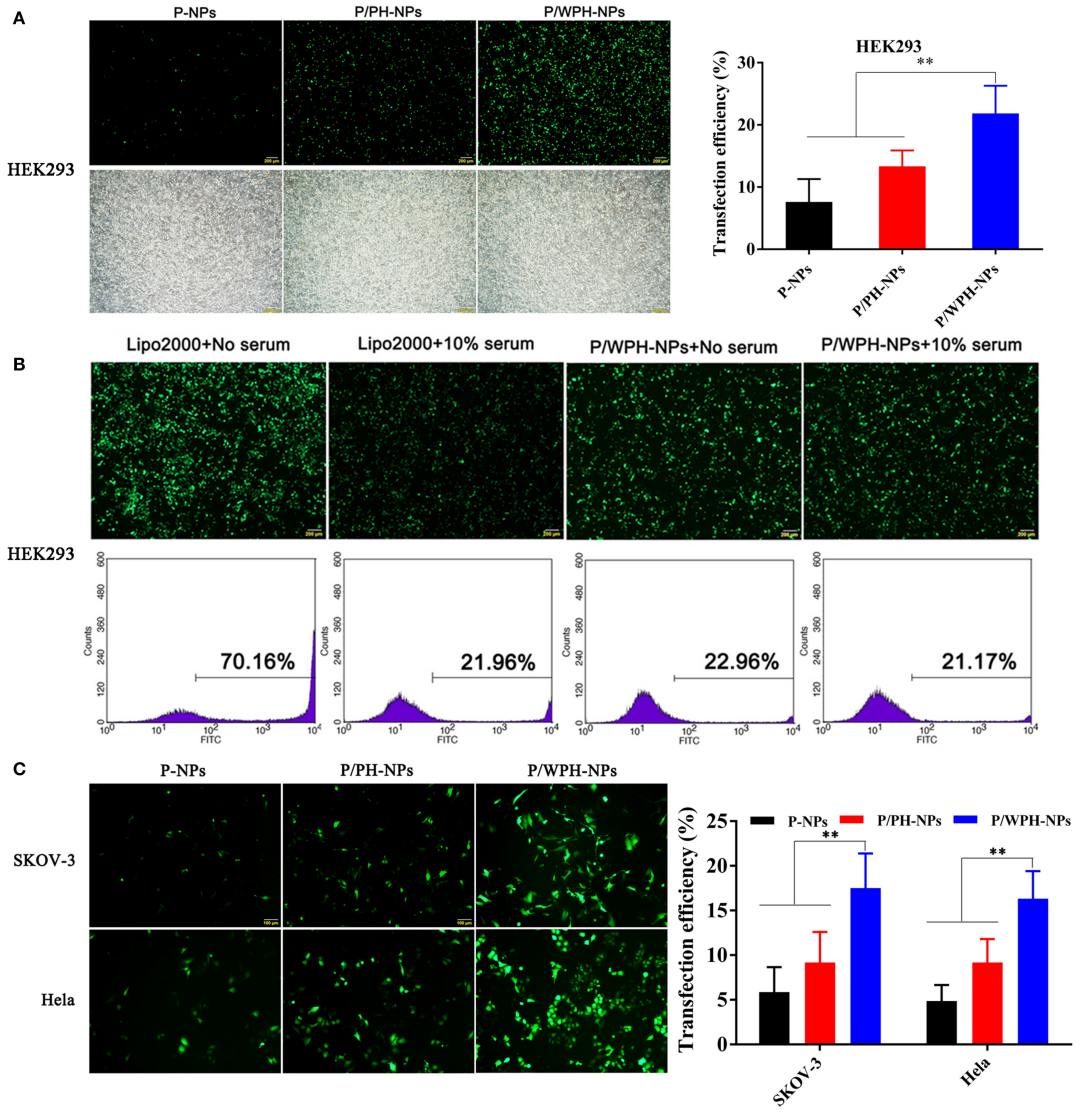
**(A)** Transfection efficiency in HEK 293 cells mediated by P-NPs, P/PH-NPs, and P/WPH-NPs. Up column: GFP fluorescence microscopy photographs; Down column: light microscopy photographs. **(B)** Effects of serum on transfection of Lipofectamine® 2000 and P/WPH-NPs on HEK 293 cells. Up column: GFP fluorescence microscopy photographs; Down column: flow cytometry graphs. **(C)** Transfection efficiency in SKOV-3 and Hela cells mediated by P-NPs, P/PH-NPs, and P/WPH-NPs. **P < 0.01.

The *in vitro* transfection activity of nanoparticles was shown in Figure 12. As present in Figures 12A,C, pGFP-P/WPH-NPs displayed the highest transfection efficiency on all the three cell lines compared with pGFP-P-NPs and pGFP-P/PH-NPs, likely because of the increased cellular uptake, fast endo/lysosome escape, elevated gene release rate, as well as the nucleus targeting property, which were surprisingly induced by the combination of the PHIS and [W5R4]K segments. Otherwise, the transfection efficiency on HEK 293 of Lipofectamine® 2000 dramatically decreases from 70.16 to 21.96% in a serum-contained medium (Figure 12B). The cause might be that the serum components especially the negatively charged proteins exhibited non-specific interactions with the cationic vector, which would largely affect the stability of the polyplex (Luan et al., 2016). However, pGFP-PLGA/WPH-NPs maintained the good transfection efficiency (22.06 vs. 21.17%) in the presence of serum, indicating that the P/WPH-NPs were quite stable and are seldom affected by the serum.

### Proliferation Inhibition of Tumor Cells of CUR/pSUR-NPs

**Figure 13:**
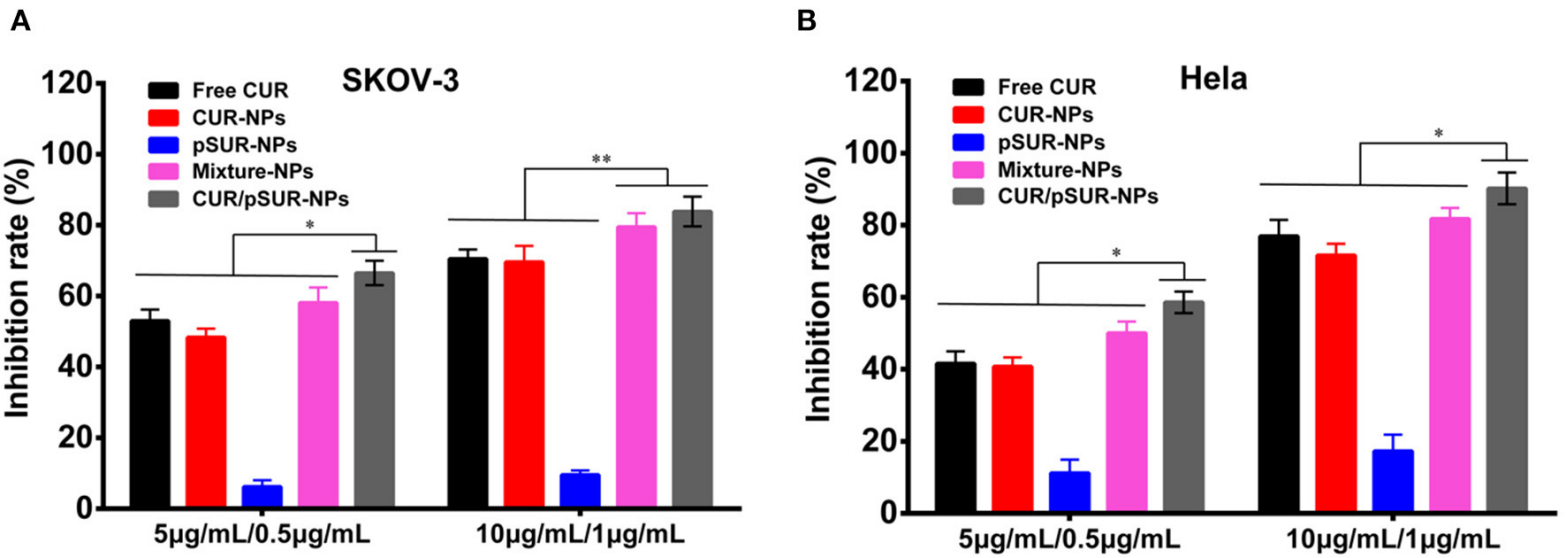
Growth inhibition of SKOV-3 **(A)** and Hela **(B)** cells after treatment with free CUR, CUR-NPs, pSUR-NPs, Mixture-NPs, and CUR/pSUR-NPs, respectively. **P < 0.01, *P < 0.05.

As shown in Figure 13, concentration-dependent cell growth inhibition activities on SKOV-3 and Hela cells of all the formulations were present. Compared to single drug- or gene-loaded nanoparticles (CUR-NPs and pSUR-NPs), CUR/pSUR-NPs, and Mixture-NPs displayed higher proliferation inhibition activity (*P* < 0.05), indicating that the anti-tumor activity could be enhanced by a combination of CUR and pSUR. Intriguingly, CUR/pSUR-NPs presented a stronger cytotoxic effect compared with Mixture-NPs (*P* < 0.05). The reason might be that Mixture-NPs would disperse their firepower against the target inevitably, because it might be difficult for Mixture-NPs to ensure the simultaneous internalization of CUR and pSUR into the same cells. Hence, encapsulating CUR/pSUR into the one nanoparticle was crucial to exert higher anti-tumor activity.

### *In vitro* Cell Apoptosis Analysis After Treatment of CUR/pSUR-NPs

**Figure 14:**
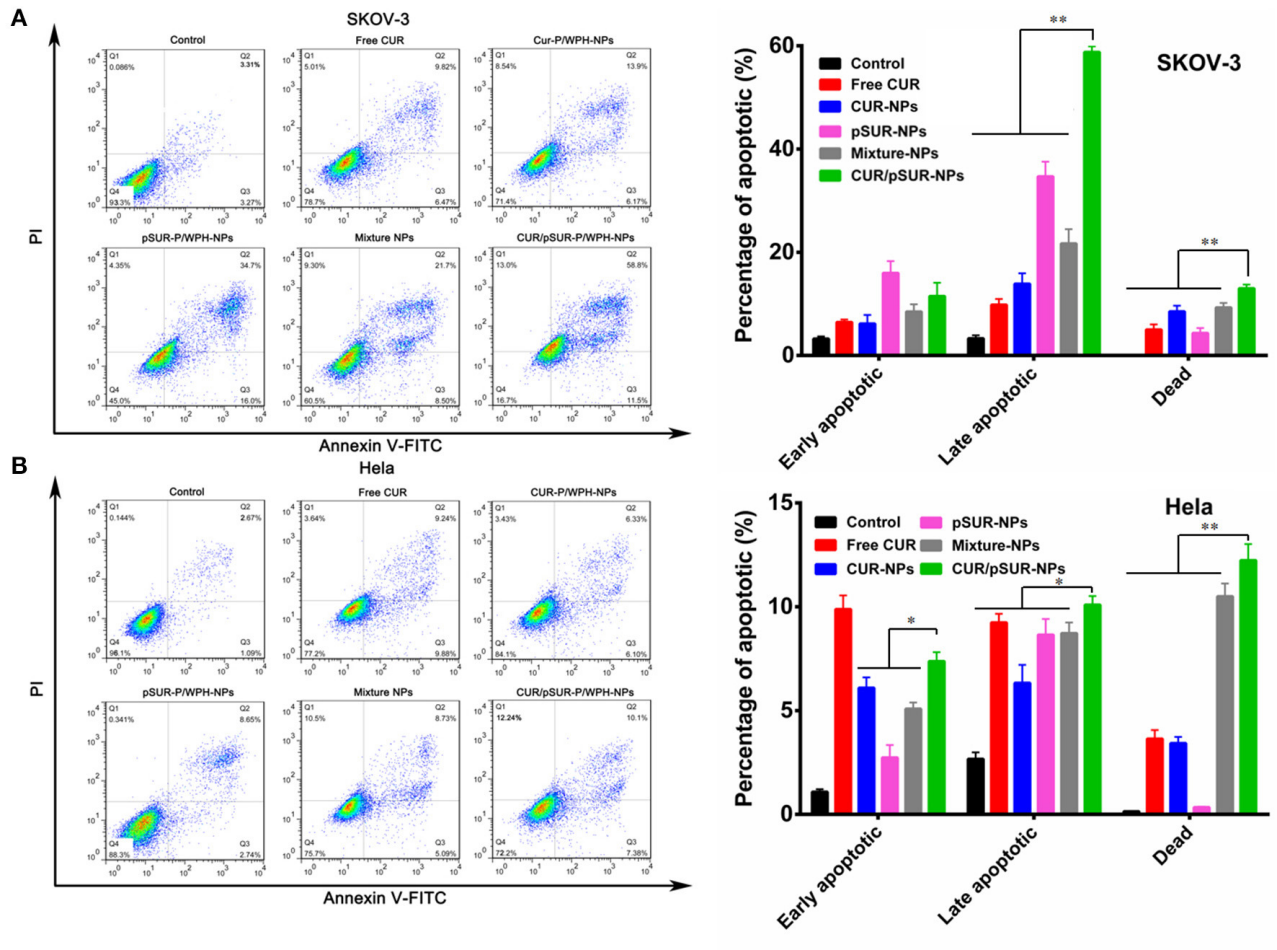
Cell apoptosis of SKOV-3 **(A)** and Hela **(B)** cells at 12 h induced by free CUR, CUR-NPs, pSUR-NPs, Mixture-NPs, and CUR/pSUR-NPs tested by flow cytometric analysis. *P < 0.05 and **P < 0.01.

Cell apoptosis induction of co-delivery and mono-delivery formulations was evaluated against SKOV-3 and Hela cells. As shown in Figure 14A, shRNA incubation increased the percentage of both early and late apoptotic SKOV-3 cells, indicating that pSUR caused apoptosis induction likely due to the downregulation of survivin protein expression. CUR/pSUR-NPs markedly increased the early apoptotic, late apoptotic, and necrotic percentages compared to the free CUR, CUR-NPs, and Mixture-NPs groups, demonstrating the synergetic anti-tumor effect of CUR and pSUR in one single nanocarrier. Additionally, the combination groups induced more necrotic cells than single drug- or gene-loaded nanoparticles (CUR-NPs and pSUR-NPs) on Hela cells (Figure 14B), implying combined therapy could induce stronger cell apoptosis effect than mono-therapy groups. Compared to the other nanoparticle formulations, CUR/pSUR-NPs had the highest proportion of early apoptotic, late apoptotic, and necrotic cells, revealing again that drug/gene co-delivery could achieve the synergistic apoptosis-inducing effects.

### Cleaved Caspase-3 Immunofluorescence Staining and Nucleus Morphology Staining

**Figure 15:**
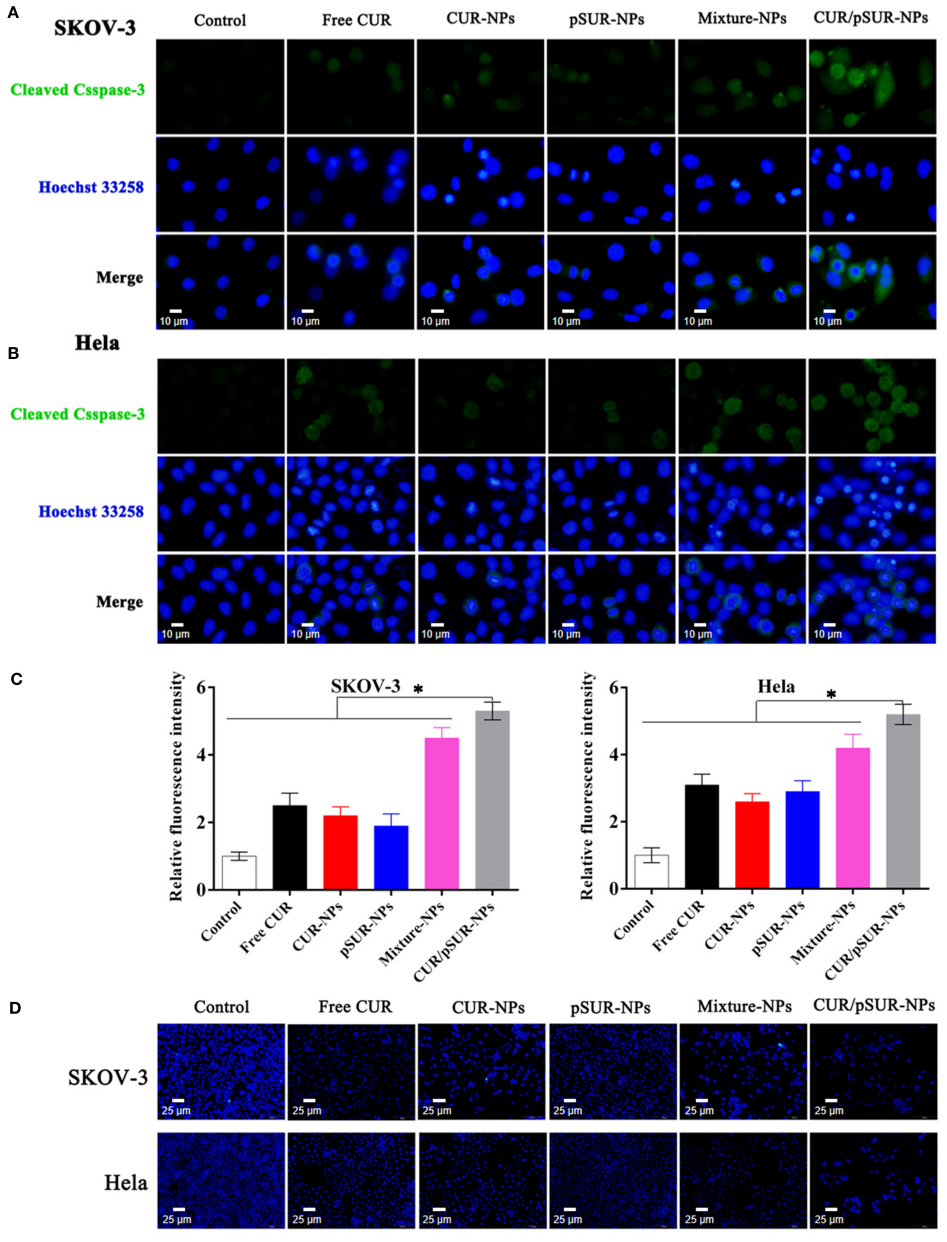
Immunofluorescence staining analysis (with 3 replicates) of cleaved caspase-3 in SKOV-3 **(A)** and Hela **(B)** cells after free CUR, CUR-NPs, pSUR-NPs, Mixture-NPs, and CUR/pSUR-NPs treatment. **(C)** Relative fluorescence intensity of cleaved caspase-3 in SKOV-3 and Hela after various formulations' treatments. **(D)** Fluorescence microscopic appearance (with 3 replicates) of Hoechst-stained nuclei of SKOV-3 and Hela cells with the treatment of free CUR, CUR-NPs, pSUR-NPs, Mixture-NPs, and CUR/pSUR-NPs. *P < 0.05.

The cysteinyl aspartate specific protease (caspase) family, which is involved in apoptotic signal transduction, plays an important role in regulating apoptosis. Caspase-3 is the final performer (Wang and Wang, 2014). Herein, to investigate caspase-3 expression after CUR/pSUR-NPs treatment, immunofluorescence staining was performed. Figures 15A,B showed that treatment of CUR, CUR-NPs, pSUR-NPs, Mixture-NPs, and CUR/pSUR-NPs significantly upregulated the expression of caspase-3 to increase apoptosis. Although caspase-3 activation was induced more strongly in the Mixture-NPs and CUR/pSUR-NPs groups, implying that the combination of CUR and pSUR was advantageous. Moreover, CUR/pSUR-NPs performed the highest fluorescence intensity, revealing transporting CUR/pSUR into the one nanoparticle was essential to exert enhanced anti-tumor activity.

Otherwise, the apoptotic rates were determined by the morphology of cancer cell nuclei which was assessed by Hoechst 33258 staining. Apoptotic or necrotic cells usually undergo chromatin margination, chromatin condensation, fragmentation, and eventual disruption (Tang et al., 2014; Hou et al., 2017). In this study, the nuclei of SKOV-3 and Hela cells treated with CUR, CUR-NPs, Mixture-NPs, and CUR/pSUR-NPs were all damaged (Figure 15C). This suggested that CUR-induced morphological changes were associated with apoptosis. CUR/pSUR-NPs treatment was the severest and many nuclei disappeared completely, pointing to the crucial need of encapsulating CUR/pSUR into one nanoparticle, which could exert a higher apoptosis rate.

## Conclusion

In summary, the newly designed copolymer WPH was synthesized for the first time, and the novel nanovector P/WPH-NPs co-encapsulating hydrophobic chemotherapy drugs (CUR) and hydrophilic therapeutic genes (pSUR) was successfully prepared (CUR/pSUR-NPs). The optimized P/WPH-NPs achieved excellent features including preferable cellular uptake, efficient endosomal escape, nuclei targeting, elevated transfection efficiency, and enhanced tumor penetration, mediated by the combined effects of PHIS and [W5R4]K. To the best of our knowledge, the tumor penetration and nuclear targeting properties of [W5R4]K conjugated to nanoparticulate formulation were first proven in our study. Moreover, CUR/pSUR-NPs exhibited a more superior anti-tumor effect than the cocktail combination of CUR-NPs and pSUR-NPs, revealing that encapsulating CUR and pSUR into the same nanoparticle and transporting them to the same tumor cell was crucial in exerting higher synergistic therapeutic efficacy. These encouraging results suggested that the P/WPH-NPs based CUR/pSUR co-delivery system would be a promising strategy for clinical application in cancer therapy.

## Data Availability Statement

The raw data supporting the conclusions of this article will be made available by the authors, without undue reservation.

## Author Contributions

This project was conceptually designed by XS and XG. BX, WZ, and LC prepared materials and carried out the majority of the experiments. Data analysis and interpretation were carried out by YZ and AF. The manuscript was prepared by BX and CJ and edited by JZ. All authors discussed the results and implications and commented on the manuscript.

## Conflict of Interest

The authors declare that the research was conducted in the absence of any commercial or financial relationships that could be construed as a potential conflict of interest.
